# Accumulation of Non-Traditional Risk Factors for Coronary Heart Disease Is Associated with Incident Coronary Heart Disease Hospitalization and Death

**DOI:** 10.1371/journal.pone.0090475

**Published:** 2014-03-13

**Authors:** Lindsay M. K. Wallace, Olga Theou, Susan A. Kirkland, Michael R. H. Rockwood, Karina W. Davidson, Daichi Shimbo, Kenneth Rockwood

**Affiliations:** 1 Department of Medicine, Dalhousie University, Halifax, Nova Scotia, Canada; 2 Department of Community Health and Epidemiology, Dalhousie University, Halifax, Nova Scotia, Canada; 3 Centre for Health Care of Elderly, QEII Health Sciences Centre, Halifax, Nova Scotia, Canada; 4 Department of Medicine, Columbia University, Presbyterian Hospital, New York, New York, United States of America; INRCA, Italy

## Abstract

**Background:**

Assessing multiple traditional risk factors improves prediction for late-life diseases, including coronary heart disease (CHD). It appears that non-traditional risk factors can also predict risk. The objective was to investigate contributions of non-traditional risk factors to coronary heart disease risk using a deficit accumulation approach.

**Methods:**

Community-dwelling adults with no known history of CHD (n = 2195, mean age 46.9±18.7 years, 51.8% women) participated in the 1995 Nova Scotia Health Survey. Three risk factor indices were constructed to quantify the proportion of deficits present in individuals: 1) a 17-item Non-Traditional Risk Factor Index (e.g. sinusitis, arthritis); 2) a 9-item Traditional Risk Factor Index (e.g. hypertension, diabetes); and 3) a frailty index (25 items combined from the other two index measures). Ten-year risks of CHD events (defined as CHD-related hospitalization and CHD-related mortality) were evaluated.

**Results:**

The Non-Traditional Risk Factor Index, made up of health deficits unrelated to CHD, was independently associated with incident CHD events over 10 years after controlling for age, sex, and the Traditional Risk Factor Index [adjusted {adj.} Hazard Ratio {HR} = 1.31; Confidence Interval {CI} 1.14–1.51]. When all health deficits, both those related and unrelated to CHD, were included in a frailty index the corresponding adjusted hazard ratio was 1.61; CI 1.40–1.85.

**Conclusion:**

Both traditional and non-traditional risk factor indices are independently associated with incident CHD events. CHD risk assessment may benefit from consideration of general health information as well as from traditional risk factors.

## Introduction

As people age, they are more likely to accumulate not just illnesses, but a variety of general health deficits which do not necessarily cross disease thresholds. Such deficits include minor impairments, performance decrements or abnormal laboratory values. People with many such health deficits are generally referred to as frail. Conceptually, frailty is a state of vulnerability characterized by loss of physiologic reserve leading to increased recovery time from environmental stressors. This leads to an accumulation of deficits going unrepaired, and can lead to adverse health outcomes [1]. Clinically, deficit accumulation can cause illness to present differently than it does in people who are otherwise healthy [2]. For example, in frail individuals, myocardial ischemia can present without chest pain, but with confusion or falls [3]. Likewise, the prognosis of myocardial infarction differs in relation to frailty [4]. An understanding of the complexity that arises from life-long deficit accumulation is integral to the clinical management of people with multiple health problems, most often older adults [2], [5]. For such reasons, the measurement of frailty has become important area of inquiry.

The frailty index approach is widely cited as a way to operationalize frailty based on deficit accumulation; it is intended to quantify the relative health state of each individual and is highly correlated with the risk of adverse health outcomes, including death [1], [6], [7], [8]. The rationale for including a range of general health deficits in an index is to consider how health outcomes may be altered by the combination of a large number of small effects [9], [10]. To date, it has been used to quantify risk for a range of adverse outcomes, including death, hospitalization, and institutionalization [6]–[8], [11]–[18]. We are now interested in understanding the role of frailty, as life-long deficit accumulation, in relation to chronic disease outcomes. Recently, a frailty index of deficits unrelated to dementia was shown to outperform traditional cognitive risk factors in the prediction of dementia [19]. This has led us to hypothesize that frailty may play an important role in the expression of late life diseases in general. Therefore, the purpose of this study was to test this hypothesis in the context of coronary heart disease (CHD) outcomes. Our specific objectives were to ask: 1) Is an index of general health deficits not traditionally associated with CHD related to incident CHD events (defined as CHD-related hospitalization, or CHD-related death)? 2) If so, does this index remain significant after controlling for established CHD risk factors? 3) Does combining all of these factors as a frailty index improve the association with incident CHD events?

## Methods

### Participants & Dataset

This analysis utilizes data from the Nova Scotia Health Survey (NSHS) undertaken in 1995 and subsequently linked to population-based medical insurance records to document incident cardiovascular events requiring hospitalization, and to vital statistics records to document deaths due to cardiovascular disease. The NSHS employed a representative probability sample designed by Statistics Canada and included 3227 non-institutionalized Nova Scotians aged 18 years and older whose names were listed in the Medical Services Insurance register. The present analysis of incident CHD events excluded participants who already had documented CHD at baseline (n = 244) and those who were missing clinic data (n = 788), leaving 2195. Demographic, anthropometric, lifestyle, and risk factor data were collected at baseline via interviewer-administered questionnaires conducted in individuals’ homes. Clinical measures were obtained by a nurse at a health care clinic. Details of the data collection are presented elsewhere [20].

The outcome was CHD-related hospitalization or CHD-related mortality over a 10 year period, reported here as CHD events. Mortality data were obtained via linkage with the National Vital Statistics database for underlying cause of death; CHD hospitalizations were obtained via linkage with the Canadian Institute for Health Information Hospital Discharge Abstract Database, which used hospital discharge summaries with a diagnostic code for ischemic heart disease within the first four positions, using the International classification of Diseases, Ninth Revision (ICD-9; 410.x through 414.x) through March 31^st^, 2005, the end of the 10 year follow up. The diagnostic codes at hospital discharge associated with medical care delivered to the study subjects provided an accurate measure for outcome assessment as Nova Scotia provides universal health care insurance, therefore reporting biases are minimized in that admission and reimbursement are not tied to the reported diagnostic code. However, people who had CHD diagnoses that did not require hospitalization or who had undiagnosed CHD would not have been considered in the outcome.

To ensure that CHD events were incident and not pre-existing, survey participants were asked about previous CHD events (heart attack, any heart problems that required surgery, or any other kind of heart problems). In addition, we reviewed discharge diagnoses for each participant for 4 years prior to the baseline survey. Survey respondents with a previous vascular disease event (documented and/or reported) were excluded from the current study sample.

Written consent was obtained from all participants, with explicit consent given for linking to health care use databases, and for the storage and future use of blood assays. Institutional review board approval was initially obtained from Dalhousie University, Halifax, Nova Scotia, Canada, and Columbia University, New York, New York, USA. Approval for the specific analyses presented here came from the Research Ethics Committee of the Capital Health District Authority, Halifax, Nova Scotia, Canada.

### Health Deficits/Measures

We created three health deficit indices, based on the deficit accumulation approach. First, we constructed a Non-Traditional Risk Factor Index (NTRFI) including 17-self-reported variables that were unrelated to coronary heart disease; these included non CHD-associated co-morbidities, activities of daily living, health conditions such as glaucoma, arthritis, sinusitis, incontinence, and dependence for personal care or affairs (see Table S1 for the full list of variables). A Traditional Risk Factor Index (TRFI) of nine variables was constructed to capture traditional risk factors for CHD as established in the literature [20], including diabetes, hypertension, and smoking (see Table S2 for the full list of variables). A third index, the frailty index, consisted of all variables included in the previously mentioned indices, except smoking. To satisfy established criteria, variables included in a frailty index can be diseases, symptoms, signs, or laboratory measures, but each should be age-related, not saturate too early (i.e. not be found in all individuals early on), be associated with adverse outcomes, and as a group, cover several bodily systems [13], [14]. Smoking was excluded from the frailty index as it violated the age-related inclusion criteria.

Each index was constructed by coding each variable as 0 or 1; 0 meaning no deficit, 1 meaning the deficit was fully represented. The index score was then calculated as the proportion of deficits present out of the total possible number of variables. For example, if a participant had 2 of the 17 deficits of the NTRFI, their score would be 2/17 = 0.12. Similarly, if the participant had 2 of the 9 deficits of the TRFI their score would be 2/9 = 0.22.

### Statistical Analysis

Descriptive statistics were used to characterize the sample and compare people with and without incident CHD events at 10-year follow-up. Having verified the assumption of proportionality, Cox proportional hazards models were used to examine the relationship each non-traditional risk factor and each traditional risk factor had with incident CHD events. We then evaluated incident CHD events in relation to the NTRFI. To further understand whether general health deficits had an independent association with incident CHD events, we did analyses examining: 1) the association between the NTRFI and incident CHD events, controlling for each individual traditional CHD risk factor in separate models, 2) the association between the NTRFI and incident CHD events in a model controlling for all individual CHD risk factors simultaneously, 3) the association between the NTRFI and incident CHD events controlling for the TRFI score. Last, to understand if the combination of all available variables was better than either the NTRFI or TRFI on its own, we tested the association between incident CHD events and the frailty index score. All regression models were adjusted for age and sex.

To test the discriminative ability of each index in predicting CHD events, we used receiver-operating characteristic (ROC) curves and evaluated the area under the curve (AUC). CHD event-free survival was examined across levels of the frailty index, using Kaplan-Meier curves.

All analyses were conducted using MATLAB (version 2007, MathWorks Inc.) and SPSS (version 15.0, SPSS Inc.). All reported confidence intervals were within 95% and statistical significance was set at a p value of 0.05.

## Results

The mean age of the sample at baseline was 46.9 years (Standard Deviation {SD} = 18.7; range 18–99). Participants who had incident CHD events during follow-up were older and more often male. The mean NTRFI for all participants was 0.10, corresponding to 1.7 deficits of a possible 17 deficits. The mean TRFI for all participants was 0.34, corresponding to 3.1 (of a possible 9) such factors present (Table 1). The mean frailty index for all participants was 0.18, corresponding to 4.5 of a possible 25 deficits. Participants that were excluded based on missing data were older, more often male, more likely to require help with personal care and had higher rates of incident CHD events at 10 years.

**Table 1:** Descriptive Characteristics of the Cohort at Baseline in Relation to Coronary Heart Disease (CHD) Event.

Variable	All participants(n = 2195)	Men(n = 1057)	Women(n = 1138)		Participants withCHD event atfollow- up (n = 174)	Participants without CHD event at follow-up (n = 1853)
Age (mean±SD^a^)	46.9±18.7	46.1±18.7		47.5±18.6	64.1±14.0	45.2±18.2*
% women	51.8	–		–	43.1	53.0*
% who need help with personal care	1.3	1.3		1.3	5.2	0.9*
% with CHD event at follow-up	7.9	9.4		6.6*	–	–
Non-Traditional Risk Factor Index (mean±SD)	0.10±0.10	0.08±0.08		0.11±0.10*	0.16±0.11	0.09±0.09*
Traditional Risk Factor Index (mean±SD)	0.34±0.19	0.35±0.19		0.33±0.20*	0.47±0.17	0.33±0.19*
Frailty index (mean±SD)	0.18±0.10	0.17±0.09		0.18±0.11*	0.27±0.10	0.17±0.10*

*p<0.05.

a SD: Standard Deviation.

During the 10-year follow up period, 174 (8%) participants had an incident CHD event (164 CHD-related hospitalizations; 10 CHD-related deaths). The mean age for experiencing an incident CHD event was 64.1 years (SD = 14.1; range 28–89). Participants who had an incident CHD event had higher average scores on all three indices relative those with no incident CHD event (Table 1).

All traditional risk factors (save physical inactivity) and four non-traditional risk factors (mental illness, chronic bronchitis/emphysema, back pain, and cancer) were associated with incident CHD events in models adjusted for age and sex (data not shown). The NTRFI was associated with incident CHD events (age and sex adjusted Hazard Ratio {adj. HR} for each 0.1 increment = 1.36, Confidence Interval {CI} 1.18–1.57, p value {p}<0.001). This association remained when adjusted for each traditional CHD risk factor individually, and in a multivariate model controlling for all individual CHD risk factors simultaneously (Table 2). It is important to note that although eight of the nine traditional risk factors were independently predictive of incident CHD events, when included in the same model, some lost significance based on the overlap of risk prediction between the variables. The association between the NTRFI and incident CHD events was also significant after controlling for all of the CHD risk factors in the TRFI (adj. HR = 1.31, CI 1.14–1.51, p = 0.001). The combined frailty index was associated with incident CHD events, and demonstrated a value for the hazard ratio farther from the null (adj. HR = 1.61, CI 1.40–1.85, p<0.001), although the confidence intervals overlapped (Table 2). Table 2 also provides unadjusted analyses and analyses only adjusted for sex to demonstrate the effects of these covariates.

**Table 2:** Cox proportional hazards model examining risk Factor Indices and Coronary Heart Disease (CHD) Risk Factors in relation to CHD events at 10 years; *p≤0.05, **p≤0.001.

Model	Variable	Unadjusted [HR (CI)]	Adjusted for sex	Adjusted for age and sex
1	Non-traditional risk factors index (per 0.1 score)	1.56 (1.36–1.79)**	1.64 (1.43–1.89)**	1.35 (1.16–1.58)**
	*Family history of cardiovascular disease	2.22 (1.50–3.28)**	2.28 (1.54–3.38)**	1.90 (1.29–2.81)**
	Hypertension	2.45 (1.75–3.44)**	2.36 (1.69–3.32)**	1.54 (1.09–2.18)*
	High low-density liproprotein	1.83 (1.29–2.61)**	1.85 (1.30–2.63)**	1.49 (1.04–2.12)*
	High triglycerides	1.36 (0.94–1.95)	1.28 (0.89–1.85)	1.50 (0.96–2.34)
	Diabetes	1.40 (0.76–2.56)	1.36 (0.74–2.50)	1.42 (0.96–2.10)
	Low high-density lipoprotein	1.05 (0.74–1.50)	1.14 (0.79–1.62)	1.28 (0.89–1.84)
	Body Mass Index	1.18 (0.77–1.82)	1.15 (0.75–1.78)	1.24 (0.67–2.27)
	Smoking	0.98 (0.67–1.43)	0.96 (0.66–1.40)	1.08 (0.76–1.54)
	Physical Inactivity	0.99 (0.71–1.38)	1.00 (0.72–1.40)	1.03 (0.74–1.43)
2	Non-traditional risk factors index (per 0.1 score)	1.55 (1.37–1.75)**	1.66 (1.46–1.88)**	1.31 (1.14–1.51)**
	Traditional risk factor index (per 0.1 score)	1.35 (1.24–1.46)**	1.34 (1.24–1.46)**	1.28 (1.17–1.39)**
3	Frailty index (per 0.1 score)	2.02 (1.79–2.72)**	2.13 (1.88–2.41)**	1.61 (1.40–1.85)**

Sensitivity analyses were undertaken by considering only the most severe CHD events as outcomes. The hazard ratios for the NTRFI (adjusted for TRFI, age, and sex) and FI (adjusted for age and sex) were 1.42, CI 1.19–1.69 and 1.75, CI 1.46–2.10 respectively when the outcome under consideration was restricted to CHD death, or hospitalization due to MI or UA only, and 1.64, CI 1.33–2.02 and 1.84, CI 1.39–2.29 respectively when the outcome under consideration was further restricted to include only the most severe events of CHD death, or hospitalization due to MI only.

**Figure 1:**
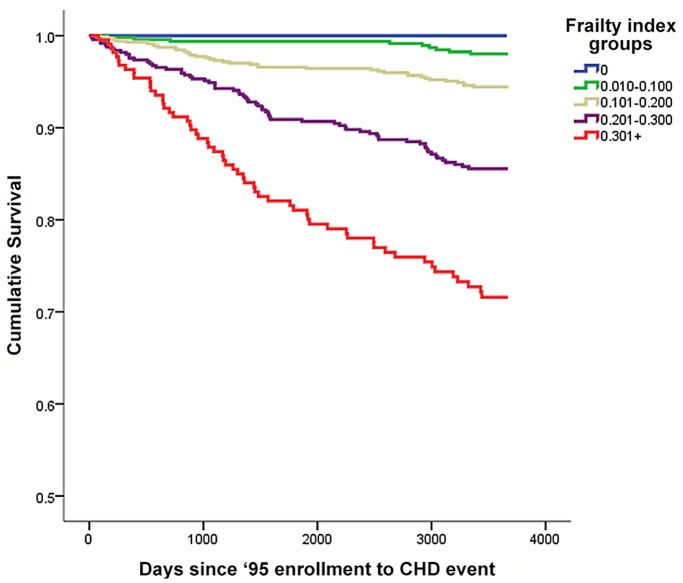
Kaplan-Meier survival curves for coronary heart disease event-free survival by levels of the frailty index.

All three indices discriminated people who had CHD events at follow-up from those who did not, with AUC scores as follows: TRFI = 0.70 (CI 0.67–0.74), NTRFI = 0.71 (0.66–0.74), frailty index = 0.76 (0.73–0.80). While the AUC score for the FI cannot be considered statistically significantly different from the TRFI or the NTRFI, the overlap in the confidence intervals was minimal. Kaplan Meier curves demonstrated that event-free survival decreased as categorized frailty index scores increased (Figure 1).

## Discussion

We investigated the impact of non-traditional risk factors for CHD as well as established (or traditional) risk factors for CHD on incident CHD events using a deficit accumulation approach. We found that an index made up of non-traditional risk factors for CHD was associated with incident CHD events, even after controlling for established risk factors individually, simultaneously, and in a comparable index score. Sensitivity analyses examining only the most severe CHD events as outcomes supported our findings.

The accumulation of deficits results in part from recovery processes becoming less efficient, leading to prolonged recovery time and increasing the likelihood of another deficit accumulating [21]. This is consistent with why the NTRFI is able to predict even specific disease outcomes like incident CHD events: deficit accumulation represents impaired damage repair processes in general, which will also operate in diseases for which specific risks are known. These findings are consistent with previous research which shows that deficit accumulation over the life course plays a role in how risk factors operate, how disease presents and progresses [4], [19], [22], and eventually how adverse health outcomes come about [14], [18], including specific disease events.

The significance of the NTRFI, made up here of factors unrelated to CHD, is that it provides a novel contribution to CHD risk stratification over and above that from established risk factors. Along these lines, a re-analysis of dementia risk factors in the Canadian Study of Health and Aging showed that traditional risk factors were greatly diminished in importance (even to the point of becoming no longer statistically significant) when “non-traditional risk factors” were considered in the prediction of dementia incidence [19]. Further, our findings are consistent with a prospective cohort study examining the relationship between cardiometabolic disease and frailty which found that frailty increased as the number of cardiometabolic disorders increased, and that frailty could stratify risk for adverse outcomes in these individuals [23]. This contributes additional evidence to the well-established cross sectional association seen between heart disease and frailty [4], [5], [11], [12], [22], [24], [25]. The consistency of this relationship suggests that risk assessment may benefit from general health information as well as from consideration of traditional risk factors.

It is important to consider that in both the traditional and non-traditional risk factor indices, each of the items is weighted equally, whereas in vascular risk factor indices, items are commonly weighted based on various multivariable statistical techniques. In any single dataset, a weighted risk factor index will virtually always outperform an unweighted index; the question is the extent to which the weights will generalize to other datasets. Weighting is a means of understanding the extent to which a given item, on average, contributes to a particular outcome. As people age, however, they need not have the same deficits to experience the adverse outcomes of deficit accumulation. Counting unweighted deficits is the most transparent way to compare the impact of risk factors. It also points to another way to understand how deficits accumulate, and why deficits accumulated in some organ systems (e.g. the musculoskeletal system) might impact deficits accumulated in other systems (e.g. the cardiovascular system). One way is that they might be directly related: inflammatory mechanisms might operate in each, for example. Another is indirect: musculoskeletal problems might impact the heart by diminishing the ability to exercise. But a third way to understand how deficits in one organ system might predispose to adverse outcomes related to another organ system is that the deficit in one system marks a more general inability of the individual to repair (or remove) damage: deficit accumulation, when the environmental exposures are constant, marks a more general slowing of recovery time. This approach to deficit accumulation can be studied formally, e.g. using queuing theory [21]. The reason to undertake such inquires is that, just as we recognize that heart disease – or other common illnesses – present differently in older adults [26] – so too does it appear that risk factors for age-related illnesses need to be considered in relation not just to age, but to the number of other things that people have wrong with them.

Our findings should be interpreted with caution. Coronary heart disease events included only those severe enough to cause hospitalization or death, and thus we may have missed patients with milder CHD events such as mild stable angina pectoris or silent MI. In consequence, the extent to which our results reflect an increased tendency to hospitalization for CHD versus CHD itself needs evaluation in future studies. It is noteworthy that our outcome measure included hospitalizations for which the discharge diagnosis summary listed an ischemic heart disease ICD code as one of the first four discharge diagnoses, indicative of either the primary or a strongly contributing factor to the hospitalization. This, coupled with the fact that participants who were documented to have CHD before or at baseline were excluded and this was the first recorded hospitalization with a CHD diagnosis, gives us confidence in identifying the outcome as incident CHD events, defined as CHD-related hospitalization or CHD-related death. The majority of hospitalizations were due to myocardial infarction (MI) or unstable angina (UA), events that are likely to be the cause, or highly related to the cause of hospitalization.

Regarding the use of the deficit accumulation approach to frailty, it has been suggested that at least 30 variables be included in the index [13]; in the present study the available number of candidate variables was limited to 25 in the FI. Despite this, the FI met established criteria and displayed characteristic features of a frailty index (increase with age, gamma distribution, higher average values for women) [14].

The deficit accumulation approach is not the only way to operationalize frailty. Another widely cited approach is the frailty phenotype, in which frailty is defined as a clinical syndrome displaying three or more of the following criteria: unintentional weight loss, exhaustion, slow walking speed, low physical activity, and weakness [27]. While the two approaches are conceptually similar, it has been shown that, at least when analyzed as a continuous variable, the frailty index can more precisely discriminate risk for death as well as measure change after an intervention [7], [28] (analyses in which it is dichotomized are less persuasive [29]). The frailty index is also less restrictive, in being evaluable by using a diverse array of information that is commonly available in clinical and epidemiological datasets.

Clinically, there is merit in modeling CHD risk using the deficit accumulation approach beyond that of added explanatory value. Patient management should reflect not just the presenting illnesses, but the overall state of health. This includes the accumulation of a range of deficits that might not cross an illness threshold [5], [30]. These results also have implications for understanding how general health deficits might increase the risk of a chronic disease, in this instance, measured as CHD-related hospitalizations and deaths. Clinically, impaired recovery time can be illustrated by considering two patients with equally severe CHD, in whom a comorbid illness such as anemia would be more likely to give rise to myocardial ischemia. In addition to a general account of factors that impair physiological reserve, the frailty index might reflect shared mechanistic factors, so that crossing a clinical threshold in one system may point to subclinical disease in another system. Recent work from an animal model of frailty and heart disease suggests that both shared disease mechanisms and impaired reserve are likely [31].

This study elucidates the independent role of an index of non-traditional risk factors for CHD in the development of CHD-related hospitalization and death, and highlights the potential usefulness of the frailty index in predicting such CHD events. This work contributes to an emerging body of research supporting the hypothesis that overall health contributes to the incidence of late-life disease. While we did not find that the frailty index was statistically significantly superior to the TRFI and NTRFI, trends in that direction were noted for each of the outcomes considered, which is motivating our group to undertake additional research in this area using increased sample sizes. Clinical tools such as the comprehensive geriatric assessment help inform how general health deficit accumulation contributes to illness as people age [18]. Further investigation into the relationship between frailty and incidence of other late-life diseases is motivating additional research by our group.

### Conclusions

Analysis of 2195 community-dwelling adults without known CHD demonstrated that an index of non-traditional risk factors for CHD could independently predict risk of CHD-related hospitalization and death. This finding suggests that CHD risk assessment may benefit from consideration of general health information as well as from traditional risk factors. Future research investigating the contributions of the frailty index in the prediction of specific disease events will provide additional insight into these complex mechanisms.

## Supporting Information

Table S1 Variables included in the Non Traditional Risk Factor Index (NTRFI).
(DOC)

Table S2 Variables included in the Traditional Risk Factor Index (TRFI).
(DOCX)
