# Supplementary material for: Accumulation of Non-Traditional Risk Factors for Coronary Heart Disease Is Associated with Incident Coronary Heart Disease Hospitalization and Death
Source: PLoS One. 2014 Mar 13;9(3):e90475. doi: 10.1371/journal.pone.0090475 (PMC3953643; doi:10.1371/journal.pone.0090475)
Supplement: Table S1 — Variables included in the Non Traditional Risk Factor Index (NTRFI). (DOC) [file pone.0090475.s001.doc]

Table S1.

| **Health deficit** | **Measurement** | **Scoring** |
| --- | --- | --- |
| Arthritis/rheumatism | Self-report | Yes=1; No=0 |
| Back problems | Self-report | Yes=1; No=0 |
| Osteoporosis | Self-report | Yes=1; No=0 |
| Chronic bronchitis/emphysema | Self-report | Yes=1; No=0 |
| Sinusitis | Self-report | Yes=1; No=0 |
| Cancer | Self-report | Yes=1; No=0 |
| Stomach/intestinal ulcers | Self-report | Yes=1; No=0 |
| Urinary incontinence | Self-report | Yes=1; No=0 |
| Cataracts | Self-report | Yes=1; No=0 |
| Glaucoma | Self-report | Yes=1; No=0 |
| Hysterectomy/oopherectomy | Self-report | Yes=1; No=0 |
| Mental illness | Self-report | Yes=1; No=0 |
| Other long-term condition | Self-report | Yes=1; No=0 |
| Need help with personal care | Self-report | Yes=1; No=0 |
| Need help with personal affairs | Self-report | Yes=1; No=0 |
| Able to go outside in good weather | Self-report | No=1; Yes, with assistance=0.5; Yes, without assistance=0 |
| Confined to a bed or chair most of day | Self-report | Yes=1; No=0 |
