# Supplementary material for: Accumulation of Non-Traditional Risk Factors for Coronary Heart Disease Is Associated with Incident Coronary Heart Disease Hospitalization and Death
Source: PLoS One. 2014 Mar 13;9(3):e90475. doi: 10.1371/journal.pone.0090475 (PMC3953643; doi:10.1371/journal.pone.0090475)
Supplement: Table S2 — Variables included in the Traditional Risk Factor Index (TRFI). (DOCX) [file pone.0090475.s002.docx]

Table S2.

| **Health deficit** | **Measurement** | **Scoring** |
| --- | --- | --- |
| Diabetes | Self-report | Yes=1; No=0 |
| Hypertension | Clinical measurement | >140 systolic and/or >90 diastolic =1; all other values =0 |
| Abnormal body-mass index | Clinical measurement | <18.5, >30=1; 25-30=0.5; 18.5-25=0 |
| High low-density lipoprotein | Clinical measurement | >3.3=1; <3.3=0 |
| Low high-density lipoprotein | Clinical measurement | <1=1; >1=0 (males),  <1.3=1; >1.3=0 (females) |
| High triglyceride level | Clinical measurement | >1.7=1; <1.7=0 |
| Family history of heart attack/angina/stroke | Self-report | Yes=1; No=0 |
| Current smoking | Self-report | Yes=1; No=0 |
| Physical inactivity | Self-report | Yes=1; No=0 |
